# Alkylating Agent-Induced Toxicity and Melatonin-Based Therapies

**DOI:** 10.3389/fphar.2022.873197

**Published:** 2022-03-23

**Authors:** Javier Egea, Francisco López-Muñoz, Oscar Fernández-Capetillo, Russel J. Reiter, Alejandro Romero

**Affiliations:** ^1^ Molecular Neuroinflammation and Neuronal Plasticity Research Laboratory, Research Unit, Hospital Universitario Santa Cristina, Instituto de Investigación Sanitaria-Hospital Universitario de La Princesa, Madrid, Spain; ^2^ Faculty of Health, University Camilo José Cela, Madrid, Spain; ^3^ Neuropsychopharmacology Unit, Hospital Doce de Octubre Research Institute (i+12), Madrid, Spain; ^4^ Genomic Instability Group, Spanish National Cancer Research Centre, Madrid, Spain; ^5^ Science for Life Laboratory, Division of Genome Biology, Department of Medical Biochemistry and Biophysics, Karolinska Institute, Stockholm, Sweden; ^6^ UTexas Health San Antonio, Long School of Medicine, San Antonio, TX, United States; ^7^ Department of Pharmacology and Toxicology, Faculty of Veterinary Medicine, Complutense University of Madrid, Madrid, Spain

**Keywords:** melatonin, alkylating agents, toxicity, inflammation, oxidative stress, molecular therapeutics, cancer, drug safety

Alkylating agents (AAs), are a group of chemo drugs generally divided into six classes; triazenes, nitrogen mustards, nitrosoureas, ethylenimine and methylenamine derivatives, alkyl sulfonates, and platinum-containing antineoplastic agents, which are commonly used, alone or in combination, in high-dose chemotherapy regimens to treat certain types of cancer (Ralhan and Kaur, 2007). These drugs undergo intramolecular cyclization reactions giving rise to highly reactive electrophilic cations, which alkylate nucleophilic sites adding an alkyl group at either oxygen, nitrogen, phosphorous or sulphur atoms on biological molecules (Soll et al., 2017). Consequently, AAs act mainly by inter- and intra-strand cross-linking DNA and RNA and hence inhibiting all cellular reactions using nucleic acid templates such as DNA replication, transcription and translation. Due to its effects on replication, the cytotoxicity induced by AAs is particularly acute in cancer cells or in certain fast-growing normal cells (hematopoietic, reproductive, and endothelial) (Fu et al., 2012). Besides DNA damage, AAs promote molecular changes that can also contribute to cell death such as oxidative stress induction (through glutathione depletion, lipid peroxidation and an overall increase in reactive oxygen and nitrogen species) (Zhang et al., 2021); or the depletion of NAD^+^ pools upon activation of the DNA repair enzyme Poly (ADP-ribose) polymerase (PARP-1) by AAs (Megnin-Chanet et al., 2010). In addition, events such as inflammation, calcium overload, mitochondrial disruption (Sahu et al., 2021), matrix metalloproteinase-9 (MMP-9) activation (Escalona et al., 2021) and alteration in NF-kB/p53/p38 MAPKs signaling pathways (Tian et al., 2019), among others, can also contribute to the overall toxicity of AAs. On the basis of this complex scenario, it is essential to find a homeostatic balance that allows for the targeted treatment of cancer cells while protecting the normal cells by reducing the side effects of AAs.

In recent years, the ubiquitous methoxyindole melatonin, has received increased attention as an adjuvant supportive treatment in patients of several cancers types (Li et al., 2017). Melatonin is a well characterized antioxidant, anti-inflammatory and free radical scavenger (Reiter et al., 2017). Because of these actions, we have previously hypothesized that melatonin would protect healthy cells from the anticancer chemotherapeutic drug mechlorethamine, by preventing the formation of the guanidine-nitrogen mechlorethamine adducts (Romero et al., 2021) and, therefore, the DNA damage. In addition, pleiotropic melatonin has been shown to protect genome integrity through the modulation of epigenetic mechanisms and recent data seems to indicate that this capacity may represent an interesting opportunity to be explored in the context of genotoxic chemotherapy of oncologic patients (Capote-Moreno et al., 2019). Furthermore, melatonin displays a large number of actions in cancer cells, either through melatonin receptors type-1 (MT1) and receptor type-2 (MT2) which mediate its antiproliferative actions, as well as by reaching intracellular organelles due its high lipophilic nature and eventually activating nuclear receptor-signaling and binding specifically to the ligand-inducible and transcriptionally active Retinoid Z receptor (RZR) or Retinoid Orphan Receptor (ROR) (Liu et al., 2016).

There is strong clinical evidence that germ cells are especially sensitive after administration of AAs, which leading to DNA damage and consequent induction of cell death (Meistrich, 2020). In this context, ovarian protection has been shown with melatonin mitigating cisplatin-induced follicle loss (Jang et al., 2016) and reducing nitro-oxidative stress and apoptosis (Moradi et al., 2021). Likewise, the use of amifostine, a radioprotective agent, in combination with melatonin, may be the “two better than one” against the production of reactive oxygen and nitrogen species (RONS) and DNA damage triggered by cisplatin in germinal epithelium cells (Eren et al., 2020). Therefore, the strategy of synergistic effect based on two antioxidants, amifostine and melatonin, could be attractive to ameliorate the AAs-induced side-effects.

Nutritional interventions may play an important role both in the growth and development of tumor cells and in the treatments with established chemotherapy regimens to maximize the drug efficacy and tolerability. Thus, it has recently been reported that metabolic changes associated with fasting has led to a reduction the toxic effects of chemotherapy while enhancing therapeutic efficacy (De Groot et al., 2020). The involvement of melatonin in this scenario is promising, since it has been well documented that melatonin regulates glucose homeostasis in peripheral tissues, from cytosolic glycolysis to mitochondrial oxidative phosphorylation (Bazwinsky-Wutschke et al., 2014; Hevia et al., 2015). It is widely known that glucose levels are fundamental for the survival and growth of many types of cancer. In this sense, has been recently documented the ability of melatonin to reduce the glucose uptake in tumor cells (Rodriguez et al., 2021). Nevertheless, the molecular and cellular mechanisms underlying melatonin´s actions need to be further exploration.

Important questions still need to be addressed regarding the combined use of chemotherapies and melatonin. When would it be reasonable to use melatonin as a supplement with a chemotherapy? Melatonin production declines with age in humans which may is thought to contribute to numerous dysfunctions and make organs more vulnerable to the development of pathologies, including cancer (Hill et al., 2013). Among others, the decline in the biosynthesis of melatonin with age has been suggested as one of the major contributors to immunosenescence and the development of neoplastic diseases (Schernhammer and Schulmeister, 2004). When melatonin is exogenously administered, all subcellular pools of melatonin are increased, with lipid-rich membranes possibly being a major reservoir for this protective molecule. As a result of its ability to enter healthy cells, it can act as a protective agent against toxic chemotherapies. The therapeutic use of this indoleamine as an adjuvant with conventional chemotherapeutic regimen could be a strategy for reducing the molecular damage to non-tumor cells resulting from conventional radio/chemotherapeutics, and enhancing the efficacy of the treatments designed to kill cancer cells.

Protecting normal cells from the side effects of chemotherapy has gained significant interest. In this regard, we recently proposed a strategy based on a combined treatment of chemotherapeutic drugs plus melatonin (Gil-Martin et al., 2019). In this context, protective profile of melatonin versus AAs has been achieved in different models (see Table 1). The hypothesis is that melatonin might limit the toxic-side effects of the chemotherapy in normal cells thereby allowing higher doses of the drugs. Importantly, a large number of studies in both animals and humans have uncovered no acute or chronic toxicities of melatonin; and the drug is widely considered to be safe even at high doses (Andersen et al., 2016; Menczel Schrire et al., 2021). This information is crucial for the clinical management of cancer patients. The optimal melatonin dosing regimens in chemotherapy-treated patients is currently under investigation, and it is possible be that the doses required may be substantially higher than those found at physiological levels, i.e., exceed those used to mitigate sleep disturbances. In this context, it is possible than an oral dose of 1 mg/kg b.w would be required.

**TABLE 1:** Summary of *in vivo* studies including melatonin as an adjuvant against AAs-induced toxicity.

References	Model	Treatment	Results
Kim et al. (2019)	C57BL/6N mice	Melatonin plus cisplatin	Melatonin ameliorates cisplatin-induced acute renal failure
Zakria et al. (2021)	Adult male white albino mice		Melatonin reduces cisplatin-induced oxidative stress and significantly improved the cognitive functions
Huang et al. (2021)	BALB/c nude tumor-bearing female mice and C57BL/6 female mice		Melatonin enhanced the anti-cancer effect of cisplatin as well as reduced ovarian toxicity
De Araujo et al. (2019)	Female Wistar rats		Melatonin decreases cisplatin-induced ototoxicity
Macit et al. (2013)	Male Sprague-Dawley rats	Melatonin plus mechlorethamine	Melatonin exerted protection against mechlorethamine-induced lung injury
Kunak et al. (2012)	Male Sprague-Dawley rats		Melatonin reduced mechlorethamine-induced kidney injury
Ilbey et al. (2009)	Male Wistar rats	Melatonin plus cyclophosphamide	Melatonin prevents cyclophosphamide -induced testicular damage
Ferreira et al. (2013)	Male Wistar rats	Melatonin plus cyclophosphamide	Melatonin blocks cyclophosphamide-induced chromosome aberrations
Cui et al. (2017)	ICR male mice	Melatonin plus busulfan	Melatonin prevents busulfan-induced testicular injury

Chemoresistance and tumor relapse represent two important challenges for increasing the quality and life expectancy of cancer patients. Tumor cells often develop resistance to several chemotherapeutic drugs through various mechanisms such as dampened apoptosis, drug efflux through ATP-binding cassette (ABC) transporters, alterations in pro-survival signaling pathways or improved DNA repair mechanism (Mollaei et al., 2021). Thus, for therapeutic scheduling several chemotherapeutic drugs may have to be used concurrently to reduce chemoresistance, increasing side effects. In this regard, certain evidences suggest that melatonin may not only reinforce the therapeutic effect of chemotherapy modalities but also reduce chemoresistance. For instance, in malignant glioma cells melatonin downregulates the overexpressed ABC transporter (Martin et al., 2013). In addition, activation of the nuclear factor erythroid 2-related factor 2 (NRF2) signaling is involved in the development of chemoresistance and melatonin upregulates NRF2 through its specific receptors MT1 and MT2, SIRT1 and PI3K/Akt pathways in non-tumor cells. In contrast, melatonin has been shown to inhibit glucocorticoid-induced kinase 1 (SGK1)-mediated NRF2 upregulation in tumor cells (Wang et al., 2019). Finally, melatonin was shown overcome cisplatin chemoresistance by inhibiting expression of Wnt/β-catenin response genes and enhancing the efficacy of this alkylant agent (Zhang et al., 2020).

A current focus of our group is to develop high dose melatonin formulations that will be beneficial for its use in cancer patients by mitigating its side-effects on normal cells and enabling a higher dosing of the chemotherapy. We are currently working on the pharmacokinetics characteristics, that should help us direct the adjuvant use of orally and/or parenterally administered melatonin in cancer patients undertreatment with AAs. Ultimately, our purpose is to help deciphering the potential of melatonin to maximize the drug efficacies while mitigating chemotherapeutic drug-induced toxicity and side effects in cancer patients.
